# Multisectoral prioritization of zoonotic diseases in Uganda, 2017: A One Health perspective

**DOI:** 10.1371/journal.pone.0196799

**Published:** 2018-05-01

**Authors:** Musa Sekamatte, Vikram Krishnasamy, Lilian Bulage, Christine Kihembo, Noelina Nantima, Fred Monje, Deo Ndumu, Juliet Sentumbwe, Betty Mbolanyi, Robert Aruho, Winyi Kaboyo, David Mutonga, Colin Basler, Sarah Paige, Casey Barton Behravesh

**Affiliations:** 1 Ministry of Health, Kampala, Uganda; 2 Zoonotic Disease Coordination Office, National One Health Platform, Kampala, Uganda; 3 United States Centers for Disease Control and Prevention, Atlanta, Georgia, United States of America; 4 Ministry of Agriculture Animal Industry and Fisheries, Entebbe, Uganda; 5 Ministry of Water and Environment, Kampala, Uganda; 6 Uganda Wildlife Authority, Kampala, Uganda; 7 Preparedness & Response Project, United States Agency for International Development, Kampala, Uganda; 8 Global Health Fellows Program II, United States Agency for International Development, Washington, DC, United States of America; Division of Clinical Research, UNITED STATES

## Abstract

**Background:**

Zoonotic diseases continue to be a public health burden globally. Uganda is especially vulnerable due to its location, biodiversity, and population. Given these concerns, the Ugandan government in collaboration with the Global Health Security Agenda conducted a One Health Zoonotic Disease Prioritization Workshop to identify zoonotic diseases of greatest national concern to the Ugandan government.

**Materials and methods:**

The One Health Zoonotic Disease Prioritization tool, a semi-quantitative tool developed by the U.S. Centers for Disease Control and Prevention, was used for the prioritization of zoonoses. Workshop participants included voting members and observers representing multiple government and non-governmental sectors. During the workshop, criteria for prioritization were selected, and questions and weights relevant to each criterion were determined. We used a decision tree to provide a ranked list of zoonoses. Participants then established next steps for multisectoral engagement for the prioritized zoonoses. A sensitivity analysis demonstrated how criteria weights impacted disease prioritization.

**Results:**

Forty-eight zoonoses were considered during the workshop. Criteria selected to prioritize zoonotic diseases were (1) severity of disease in humans in Uganda, (2) availability of effective control strategies, (3) potential to cause an epidemic or pandemic in humans or animals, (4) social and economic impacts, and (5) bioterrorism potential. Seven zoonotic diseases were identified as priorities for Uganda: anthrax, zoonotic influenza viruses, viral hemorrhagic fevers, brucellosis, African trypanosomiasis, plague, and rabies. Sensitivity analysis did not indicate significant changes in zoonotic disease prioritization based on criteria weights.

**Discussion:**

One Health approaches and multisectoral collaborations are crucial to the surveillance, prevention, and control strategies for zoonotic diseases. Uganda used such an approach to identify zoonoses of national concern. Identifying these priority diseases enables Uganda’s National One Health Platform and Zoonotic Disease Coordination Office to address these zoonoses in the future with a targeted allocation of resources.

## Introduction

Zoonoses now represent approximately 70% of newly emerging diseases [1]. Additionally, new and re-emerging zoonotic diseases have resulted in negative socioeconomic impacts, affecting national policies that range from health security to the control of trans-boundary animal diseases [2]. Their impacts manifest in multiple ways, including animal illness and loss of productivity, loss of income for livestock-dependent populations, and human morbidity and mortality [2].

Uganda is particularly vulnerable to zoonotic diseases due to its unique biological diversity and rising population density, bringing humans and animals into increasing and more intensive contact [3–5]. Additionally, over 80% of Uganda’s population is engaged in agriculture with 58% of these individuals involved in livestock farming [3]. Uganda has an estimated 14.3 million cattle, 15.7 million goats, 4.3 million sheep, 4 million pigs, and 46.2 million poultry [6]. Multiple outbreaks of highly pathogenic zoonotic diseases including Ebola, Marburg, and Rift Valley fever have occurred in Uganda since 2008 [7,8].

On November 3, 2016, the Government of Uganda launched a National One Health Platform (NOHP) to spearhead collaborative efforts amongst four government sectors to prevent, detect, and respond to existing zoonotic diseases as well as emerging pandemic threats. The government sectors include the Ministry of Health; the Ministry of Agriculture, Animal Industries, and Fisheries; the Ministry of Water and the Environment; and the Uganda Wildlife Authority. The platform is comprised of two institutional structures–the One Health Technical Working Group (OHTWG), which provides oversight and direction, and the Zoonotic Disease Coordination Office (ZDCO), which serves as the secretariat of the platform.

The OHTWG is co-chaired by technical directors of the collaborating ministries on a six-month, rotational basis and has 36 members representing core government One Health sectors and partner organizations including United Nations agencies, United States Government agencies, academia, Uganda Veterinary and Medical Associations, research institutions, and other technical partners. The secretariat is comprised of eight staff members (two from each sector) who act as sector focal persons but hold positions in their respective ministries. This structure integrates technical expertise with government institutions, resulting in policy development that combines scientific evidence with political capacity.

To more effectively address zoonotic disease challenges in Uganda, we conducted a One Health Zoonotic Disease Prioritization (OHZDP) workshop. The purpose of the two-day workshop was to use a multisectoral, One Health approach to identify and prioritize zoonotic diseases of greatest national concern while also facilitating the development of zoonoses-specific multisectoral disease control and prevention strategies in Uganda. This article details the semi-quantitative One Health approach used to prioritize zoonoses in Uganda.

## Materials and methods

We conducted the zoonotic disease prioritization through a facilitated-consultative process involving 35 zoonoses experts representing human, animal, and environmental health ministries as well as key partners who observed the workshop (Table 1). Nine participants were chosen prior to the workshop to serve as voting members and represented key government ministries involved in addressing zoonoses. Voting members were chosen by their respective ministries after invitations were sent requesting zoonoses experts. Non-voting participants (observers) from non-governmental organizations, academia, and research were invited based on their organization’s involvement in addressing zoonoses (Table 1). All attendees voluntarily agreed to participate. The workshop was conducted March 2–3, 2017 in Kampala, Uganda.

**Table 1:** The Uganda One Health Zoonotic Disease Prioritization Workshop Participating organizations–Kampala, Uganda, 2017.

Participating Organization
Coordination Office for the Control of Trypanosomiasis in Uganda
Food and Agriculture Organization
Infectious Disease Institute
Makerere University
One Health East and Central Africa
Uganda Medical Association
Uganda Ministry of Agriculture, Animal Industry and Fisheries*
Uganda Ministry of Health*
Uganda Ministry of Water and Environment*
Uganda National Animal Disease Diagnostics and Epidemiology Centre
Uganda National Institute for Public Health
Uganda National Laboratory Health Services
Uganda One Health Technical Working Group
Uganda Public Health Emergency Operation Centre
Uganda Veterinary Association
Uganda Virus Research Institute
Uganda Wildlife Authority*
Uganda Zoonotic Disease Coordination Office
United States Agency for International Development
World Health Organization

* Representatives from these organizations served as voting members

### Selection of a prioritization tool

Comprehensive quantitative data on most zoonoses in Uganda is limited. In 2014, the U.S. Centers for Disease Control and Prevention (CDC) developed a OHDZP tool to be used in situations where comprehensive quantitative data is not available. This semi-quantitative tool has been described in detail elsewhere [9,10] and was selected for Uganda’s zoonoses prioritization process. The tool consists of the following steps: identification of zoonoses to be prioritized, development of five criteria to prioritize diseases, development of questions with categorical answers for each criterion, reviewing available data to answer each question, weighting of the criteria, and ranking of the zoonoses using a decision tree analysis.

In order to build in-country capacity to conduct future OHZDP workshops, CDC trained local partners to be facilitators. The local partners then served as co-facilitators during the OHZDP in Uganda with assistance from CDC facilitators. This expertise can be used for future workshops independent of external organizational assistance.

### Selection of the initial list of Uganda’s zoonotic diseases of concern and literature review

Before the workshop, a list of 48 zoonotic diseases relevant to Uganda was developed with input from animal, human, and environmental sectors as well as subject matter experts. To be included on the list, each zoonotic disease had to have known transmission in Uganda or bordering countries.

To answer questions developed for disease prioritization, we searched for literature on human and animal disease burden for the 48 selected zoonoses through NCBI PubMed and Google Scholar. Data publicly available on websites of the World Health Organization (WHO), the World Organization for Animal Health (OIE), the United Nations Food and Agriculture Organization (FAO), the CDC, ProMed, Health Map, and other relevant sites were also reviewed. If disease information for a particular zoonotic disease was not available for Uganda, data for other East or Central African countries were used. Global disease data were used when regional data were not available. Ugandan government ministries were also contacted for information not publicly available. Workshop participants filled data gaps based on expert and consensus opinions. The literature search was not a comprehensive review of the literature, but a focused search to answer questions developed during the workshop.

### Selection of criteria and weighting for Ugandan zoonotic diseases

Voting members with input from workshop observers collectively identified five criteria for quantitatively ranking the 48 zoonotic diseases. Then, each voting member individually indicated his or her preference for the relative importance of each criterion through an analytical hierarchal process [11]. This process generated a final list of weights for each criterion. The criteria selected to prioritize zoonotic diseases in order of importance were (1) severity of disease in humans in Uganda, (2) availability of effective control strategies, (3) potential to cause an epidemic or pandemic in humans or animals, (4) social and economic impacts, and (5) bioterrorism potential (Table 2).

**Table 2:** Ranking criteria and weights, associated categorical questions, and response options used for prioritizing zoonotic diseases in Uganda.

Criteria (Weighted Scores)	Question	Response and categories (score)	
Severity of disease in humans (0.21)	What is the case fatality rate in humans?	< or = 5%	(0)
		> 5%	(1)
Availability of effective control strategies (0.21)	Is there an effective control strategy in both animals and humans in Uganda?	None	(0)
		Either	(1)
		Both	(2)
Potential to cause an epidemic or pandemic in humans or animals (0.21)	Has this disease caused an epidemic in animals or humans in the last 10 years in Uganda?	None	(0)
		Either	(1)
		Both	(2)
Social and economic impacts (0.19)	Does the disease reduce animal productivity by 10% or more?	No	(0)
		Yes	(1)
Bioterrorism potential (0.19)	Is the disease listed as a select agent by USDA/HHS?	No	(0)
		Yes	(1)

### Question selection for each criterion

Using group discussion, voting members developed questions with categorical answers to address the criteria developed in the prior step. Data generated from the focused literature review or from expert opinion for each of the 48 zoonotic diseases were used to answer these questions (Table 2). The questions had either binomial (yes/no) or multinomial (1–5%, 5–10%, 10–20%, etc.) answers. Answer choices were assigned scores by voting members to determine which answer received the full weight of the question. These answer characteristics are necessary for the scoring process and were guided by group discussion and expert opinion (Table 2).

### Decision tree, disease weighting, and final ranking

Country-specific, regional, or global data along with expert opinion were used to determine appropriate responses for each question for all 48 zoonoses. Voting members reviewed the response to each question for all zoonoses and agreed on the scoring procedure (S1 Table). All information gaps were filled by expert opinion and consensus of workshop participants. Based on an analytical hierarchical process, a decision tree was designed in Microsoft Excel and used to calculate disease rankings. The weighted criterion and question responses were applied to each zoonotic disease to calculate a final disease score between 0 and 1. Final scores were a sum of the scores from each question. The scores for all diseases were then normalized to the highest scoring disease, which received a score of one. All workshop participants reviewed disease ranking results, which facilitated further discussion. The voting members then collaboratively finalized priority zoonotic diseases for Uganda using these results.

### Sensitivity analysis

We assessed variability in criteria weighting to determine the robustness of the prioritization outcome. First, we assigned the five selected criteria equal weights and assessed how normalized disease scores compared to weighted disease scores. We also systematically removed each of the five developed criteria and assessed normalized disease scores with the four remaining criteria. Because criteria weighting was done anonymously, we were unable to assess normalized scores by government sector. Pearson’s product-moment correlation was used to assess the relationship between normalized disease scores with a coefficient p-value <0.05 considered significant. The analysis was conducted in RStudio version 3.4 (RStudio, USA).

## Results

The criteria and weighting utilized to create the ranked list of zoonotic diseases are provided in Table 2. A full list of zoonoses by normalized score is available in Table 3. Unweighted scores assigned to each disease by criterion is provided in S1 Table. The following is a discussion of the criteria and the prioritized diseases.

**Table 3:** Normalized scores for zoonotic diseases in Uganda.

Disease	Normalized Final Score
Anthrax	1.000
Zoonotic influenza viruses	1.000
Ebola viruses	0.794
Brucellosis	0.791
Rift Valley fever (RVF)	0.791
African trypanosomiasis	0.711
Plague	0.704
Crimean Congo hemorrhagic fever (CCHF)	0.704
Rabies	0.621
Marburg	0.601
Salmonellosis	0.501
Q-fever	0.482
Listeriosis	0.402
Leptospirosis	0.399
Zoonotic tuberculosis	0.399
Bovine cysticercosis	0.399
Hydatidosis	0.399
Porcine cysticercosis	0.399
Newcastle disease	0.399
Orf (contagious ecthyma)	0.399
Tularemia	0.396
Spotted fevers	0.312
Tetanus	0.312
Leishmaniasis	0.312
MERS	0.312
Cryptosporidiosis	0.296
Toxoplasmosis	0.296
West Nile virus	0.296
Lassa fever	0.290
Prions	0.209
Ehrlichiosis	0.206
Trichnellosis	0.206
Tungiasis	0.206
Onchocerciasis	0.206
Chikungunya	0.206
Hepatitis E virus	0.206
Yellow fever	0.206
Sarcoptic mange	0.206
Bartonellosis	0.103
Campylobacteriosis	0.103
Erysipeloid	0.103
*Escherichia coli*	0.103
*Streptococcus suis*	0.103
Tick borne relapsing fever	0.103
Schistosomiasis	0.103
Dengue fever	0.103
Hantaviruses	0.103
Zika virus	0.103

Among the criteria, diseases that cause a heavy burden in human populations were considered the most important criterion by voter weighting. However, mortality data for all considered zoonoses in Uganda were not available. Additionally, mortality rates vary by population and by access to treatment. As a result, we used a proxy agreed upon by workshop participants whereby diseases should have a clear case fatality rate >5% assuming a healthy population and access to treatment. We used disease information from CDC, WHO, and OIE to answer this question. Diseases with a high mortality rate (CFR > 5%) received the full weight score of 1. These diseases included anthrax and viral hemorrhagic fevers. Diseases with a lower mortality rate (CFR < 5%) received a score of 0, which characterizes most of the non-priority diseases.

The ability to control a zoonotic disease was the second most important criterion selected in the workshop. We defined control strategies as those diseases where an effective vaccine, treatment, or vector control program exists. Information from published literature, CDC, OIE, and WHO as well as expert opinion was used to answer this question. Diseases where an effective control strategy to prevent a zoonotic disease for both humans and animals was available in the country were assigned a score of 2. Diseases for which an effective control strategy for either animals or humans was available, but not both, were assigned scores of 1. In this scenario, a control strategy for either humans or animals carried the same scoring weight. Finally, diseases with no effective control strategy in humans or animals received a score of 0, which applied to most of the viral zoonotic diseases selected.

The third most important criterion was whether a disease caused a pandemic or epidemic in humans or animals in Uganda in the prior 10 years. We used outbreak reports from WHO, OIE, and ProMED as well as expert opinion to identify previous outbreaks in Uganda [12–14]. Diseases that caused an epidemic in Uganda in both humans and animals received the full weight score of 2. If a disease caused an epidemic in either humans or animals, but not both, the disease was assigned a score of 1. Finally, diseases that had not caused an epidemic in Uganda were given a score of 0.

The fourth criterion was whether a disease reduced animal productivity. If the zoonotic disease reduced animal productivity by 10% or more, the disease received the full weight score of 1. If the zoonotic disease reduced animal productivity by less than 10%, a score of 0 was assigned. Because data were not available for many diseases in Uganda, we used the OIE reportable disease list and expert opinion to answer this question [15]. Since many of the top prioritized zoonoses have significant impacts on animal productivity, they received the full weight score of this criterion.

The potential of the disease to be used for bioterrorism was the fifth criterion. If the zoonotic disease was included in the United States Department of Health and Human Services and the United States Department of Agriculture Select Agents and Toxins List the disease was given the full weight score of 1 [16]. If the disease was not included on this list, it was given a score of 0.

During the presentation of the preliminary prioritized zoonoses list, workshop participants reviewed the question scoring of each disease. All voting members agreed on any scoring changes and agreed on the final question scoring. A final list of zoonoses by score is provided in Table 3.

Based on the list of normalized scores, the workshop participants selected diseases ranked in the top ten zoonotic diseases to be the focus of One Health efforts. The group then consolidated hemorrhagic fevers into one disease category to arrive at 7 overall prioritized zoonotic diseases. The seven zoonoses included anthrax, zoonotic influenza viruses, viral hemorrhagic fevers (Ebola, Rift Valley fever, Crimean Congo hemorrhagic fever, and Marburg), brucellosis, African trypanosomiasis, plague, and rabies (Table 4).

**Table 4:** Final prioritized disease rankings from the Ugandan One Health Zoonotic Disease Prioritization Workshop, 2017.

Disease	Final Ranking
Anthrax	1
Zoonotic influenza viruses	2
Viral hemorrhagic fevers (Ebola, Marburg, CCHF and RVF)	3
Brucellosis	4
African trypanosomiasis	5
Plague	6
Rabies	7

**Fig 1:**
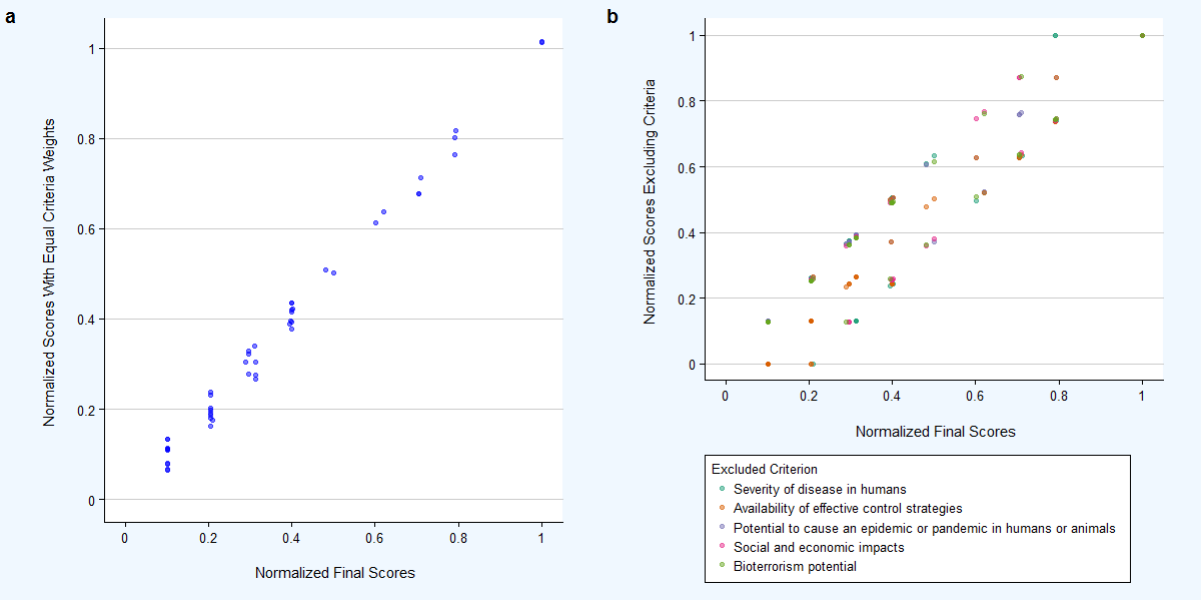
Comparison of disease prioritization scores obtained from weighted criteria and (a) equal criteria weights or (b) excluding each of the five criteria.

The sensitivity analysis showed a strong positive correlation between scores produced by the OHZDP tool and normalized disease scores using unweighted criteria (r = 0.99, p <0.01) (Fig 1A). There was also a strong positive correlation when excluding each criteria, then comparing disease scores to those produced by the OHZDP tool (r = 0.91–0.97, p <0.01) (Fig 1B). Additionally, there were few changes in the top ten zoonoses when excluding criteria (S2 Table). When excluding severity of disease in humans or the potential to cause an epidemic or pandemic in humans or animals, Q-fever was ranked 10^th^ replacing rabies. When bioterrorism potential was excluded, salmonellosis replaced Marburg as 10^th^.

## Discussion

One Health Zoonotic Disease Prioritization Workshops have now been conducted in multiple countries, generating a unique list for each country including Uganda. Using a semi-quantitative approach, Uganda was able to select priority zoonotic diseases while incorporating multisectoral input. The final list of priority zoonotic diseases in Uganda in descending order of importance was anthrax, zoonotic influenza viruses, viral hemorrhagic fevers, brucellosis, African trypanosomiasis, plague, and rabies. This list showed some commonalities and differences with results of other zoonotic disease prioritization workshops.

The top five priority zoonotic diseases in neighboring Kenya (anthrax, trypanosomiasis, rabies, brucellosis, and Rift Valley fever), were all included in Uganda’s list of seven priority zoonotic diseases [17]. However, in contrast to Kenya, Uganda chose to rank all viral hemorrhagic fevers together (Ebola, Marburg, Crimean Congo hemorrhagic fever, and Rift Valley fever) and included plague on its list [17]. In Ethiopia, the final list of prioritized diseases also included brucellosis, rabies, and anthrax, reflecting the common importance of these three diseases to both Ethiopia and Uganda [18]. Yet, whereas Ethiopia ranked leptospirosis and echinococcosis on their final list, Uganda did not include these diseases as priorities [18]. Themes from zoonotic disease prioritization workshops in seven countries from 2014–2016 were recently published [19]. Five countries prioritized rabies, zoonotic influenza viruses, and brucellosis; four anthrax; and three hemorrhagic fevers and salmonellosis.

The process of zoonotic disease prioritization allows a country to periodically reassess diseases of importance and the direction of resource allocation. The disease list produced during this process is not static and can be reviewed regularly to incorporate new data, especially as new diseases emerge in Uganda’s diverse ecosystems. The workshop also encouraged multisectoral collaboration to effectively implement prevention and control strategies.

Following the prioritization of zoonotic diseases, the workshop participants discussed recommendations for multisectoral development of laboratory capacity, surveillance, joint outbreak response activities, and prevention and control strategies to address the prioritized zoonotic diseases. Workshop participants advocated for both the mapping of current laboratory capacity, a critical need in Uganda, as well as mobilizing external resources to improve capacity.

The following items were identified as crucial to surveillance: (1) create standardized case definitions for prioritized zoonotic diseases in animals and people, (2) ensure that the prioritized zoonotic diseases are included in the mandatory list of reportable diseases in all relevant sectors, and (3) utilize the Zoonotic Disease Coordination Office as a common platform for reporting and sharing data on zoonotic diseases in humans and animals. Participants identified the need to update preparedness and response plans that incorporate a One Health approach as well as new plans for diseases that currently lack plans to improve capacity to respond to outbreaks. Finally, to enhance prevention and control activities for the prioritized zoonoses, the workshop participants recommended the following: (1) strengthen multisectoral communication and information sharing, (2) develop a national One Health strategic plan for Uganda including how to address the prioritized zoonotic diseases, and (3) outline current research efforts and research needs for the prioritized zoonoses across all relevant sectors.

Several limitations related to the use of the OHZDP tool were present in this work. First, there was a lack of strong country-specific data for many diseases, which required the use of regional or global data as well as expert opinion. The use of regional or global data may not have been an accurate measure of the impact of these diseases in Uganda. Second, the selection of criteria for prioritization may be specific to the workshop participants. Finally, the questions chosen for the tool by workshop participants may not describe the impact of all zoonoses.

## Conclusions

The OHZDP tool provides a semi-quantitative framework for prioritizing country-specific, zoonotic diseases of importance. Uganda used the tool to identify seven zoonoses of greatest national concern and to lay the foundation for multisectoral collaboration. The OHZDP tool also provided Uganda with a framework for next steps to begin addressing the prioritized zoonotic diseases. Consequent to this workshop, Uganda’s National One Health Platform will serve as the overarching body for spearheading planning and coordinating One Health activities between sectors.

## Disclosures

The findings and conclusions in this report are those of the authors and do not necessarily represent the official position of the United States Centers for Disease Control and Prevention or the United States Agency for International Development.

## Supporting information

S1 Table Scores assigned to each disease by criterion before weighting. ^Scores obtained by literature review or by expert opinion and consensus from workshop participants.
(DOCX)

S2 Table Disease rankings after sensitivity analysis. ^#^Final prioritized disease list for Uganda was selected by group discussion after overall results presented.
(DOCX)
