# Supplementary material for: Multisectoral prioritization of zoonotic diseases in Uganda, 2017: A One Health perspective
Source: PLoS One. 2018 May 1;13(5):e0196799. doi: 10.1371/journal.pone.0196799 (PMC5929520; doi:10.1371/journal.pone.0196799)
Supplement: S1 Table — ^Scores obtained by literature review or by expert opinion and consensus from workshop participants. (DOCX) [file pone.0196799.s001.docx]

**S1 Table. Scores assigned to each disease by criterion before weighting^**

| **Disease** | **Severity of disease in humans** | **Potential to cause an epidemic or pandemic in humans or animals** | **Availability of effective control strategies** | **Social and economic impacts** | **Bioterrorism potential** |
| --- | --- | --- | --- | --- | --- |
| Anthrax | 1 | 2 | 2 | 1 | 1 |
| Bartonellosis | 0 | 0 | 1 | 0 | 0 |
| Brucellosis | 0 | 2 | 2 | 1 | 1 |
| Campylobacteriosis | 0 | 0 | 1 | 0 | 0 |
| Ehrlichiosis | 0 | 0 | 2 | 0 | 0 |
| Erysipeloid | 0 | 0 | 1 | 0 | 0 |
| *Escherichia coli* | 0 | 0 | 1 | 0 | 0 |
| Leptospirosis | 0 | 0 | 2 | 1 | 0 |
| Listeriosis | 1 | 0 | 0 | 1 | 0 |
| Plague | 1 | 1 | 2 | 0 | 1 |
| Q-fever | 0 | 0 | 1 | 1 | 1 |
| Salmonellosis | 0 | 2 | 1 | 1 | 0 |
| Spotted fevers* | 1 | 0 | 1 | 0 | 0 |
| *Streptococcus suis* | 0 | 0 | 1 | 0 | 0 |
| Tetanus | 1 | 0 | 1 | 0 | 0 |
| Tick borne relapsing fever | 0 | 0 | 1 | 0 | 0 |
| Tularemia | 1 | 0 | 0 | 0 | 1 |
| Zoonotic tuberculosis | 0 | 0 | 2 | 1 | 0 |
| Bovine cysticercosis | 0 | 0 | 2 | 1 | 0 |
| Cryptosporidiosis | 0 | 0 | 1 | 1 | 0 |
| Hydatidosis | 0 | 0 | 2 | 1 | 0 |
| Leishmaniasis | 1 | 0 | 1 | 0 | 0 |
| Porcine cysticercosis | 0 | 0 | 2 | 1 | 0 |
| Schistosomiasis | 0 | 0 | 1 | 0 | 0 |
| Trypanosomiasis | 1 | 1 | 2 | 1 | 0 |
| Toxoplasmosis | 0 | 0 | 1 | 1 | 0 |
| Trichnellosis | 0 | 0 | 2 | 0 | 0 |
| Tungiasis | 0 | 1 | 1 | 0 | 0 |
| Onchocerciasis | 0 | 1 | 1 | 0 | 0 |
| Chikungunya | 0 | 1 | 1 | 0 | 0 |
| Crimean Congo Hemorrhagic Fever (CCHF) | 1 | 1 | 2 | 0 | 1 |
| Dengue Fever | 0 | 0 | 1 | 0 | 0 |
| Ebola viruses | 1 | 1 | 1 | 1 | 1 |
| Hantaviruses | 0 | 0 | 1 | 0 | 0 |
| Hepatitis E virus | 0 | 1 | 1 | 0 | 0 |
| Lassa Fever | 0 | 0 | 1 | 0 | 1 |
| Marburg | 1 | 1 | 1 | 0 | 1 |
| MERS | 1 | 0 | 1 | 0 | 0 |
| Newcastle disease | 0 | 0 | 2 | 1 | 0 |
| Orf (contagious ecthyma) | 0 | 1 | 1 | 1 | 0 |
| Rabies | 1 | 2 | 2 | 0 | 0 |
| Rift Valley fever | 0 | 2 | 2 | 1 | 1 |
| West Nile virus | 0 | 0 | 1 | 1 | 0 |
| Yellow Fever | 0 | 1 | 1 | 0 | 0 |
| Zika virus | 0 | 0 | 1 | 0 | 0 |
| Zoonotic Influenza viruses | 1 | 2 | 2 | 1 | 1 |
| Sarcoptic mange | 0 | 1 | 1 | 0 | 0 |
| Prions | 1 | 0 | 0 | 0 | 0 |

**^**Scores obtained by literature review or by expert opinion and consensus from workshop participants
