# Supplementary material for: Multisectoral prioritization of zoonotic diseases in Uganda, 2017: A One Health perspective
Source: PLoS One. 2018 May 1;13(5):e0196799. doi: 10.1371/journal.pone.0196799 (PMC5929520; doi:10.1371/journal.pone.0196799)
Supplement: S2 Table — #Final prioritized disease list for Uganda was selected by group discussion after overall results presented. (DOCX) [file pone.0196799.s002.docx]

**S2 Table. Disease rankings after sensitivity analysis**

| **Rank** | **Overall^#^** | **Equal criteria weights** | **Excluded criterion** | | | | |
| --- | --- | --- | --- | --- | --- | --- | --- |
|  |  |  | **Severity of disease in humans** | **Potential to cause an epidemic or pandemic in humans or animals** | **Availability of effective control strategies** | **Social and economic impacts** | **Bioterrorism potential** |
| 1 | Anthrax | Anthrax | Anthrax | Anthrax | Anthrax | Anthrax | Anthrax |
| 2 | Zoonotic Influenza viruses | Zoonotic Influenza viruses | Brucellosis | Zoonotic Influenza viruses | Zoonotic Influenza viruses | Zoonotic Influenza viruses | Zoonotic Influenza viruses |
| 3 | Ebola viruses | Brucellosis | Rift Valley fever | Ebola viruses | Ebola viruses | Plague | Trypanosomiasis |
| 4 | Brucellosis | Ebola viruses | Zoonotic Influenza viruses | Trypanosomiasis | Brucellosis | Crimean Congo Hemorrhagic Fever | Rabies |
| 5 | Rift Valley fever | Rift Valley fever | Ebola viruses | Plague | Rift Valley fever | Rabies | Ebola viruses |
| 6 | Trypanosomiasis | Plague | Trypanosomiasis | Crimean Congo Hemorrhagic Fever | Trypanosomiasis | Ebola viruses | Brucellosis |
| 7 | Plague | Trypanosomiasis | Salmonellosis | Brucellosis | Plague | Marburg | Rift Valley fever |
| 8 | Crimean Congo Hemorrhagic Fever | Crimean Congo Hemorrhagic Fever | Plague | Rift Valley fever | Crimean Congo Hemorrhagic Fever | Brucellosis | Plague |
| 9 | Rabies | Marburg | Crimean Congo Hemorrhagic Fever | Marburg | Marburg | Rift Valley fever | Crimean Congo Hemorrhagic Fever |
| 10 | Marburg | Rabies | Q-fever | Q-fever | Rabies | Trypanosomiasis | Salmonellosis |
| 11 | Salmonellosis | Q-fever | Rabies | Rabies | Listeriosis | Tularemia | Marburg |
| 12 | Q-fever | Salmonellosis | Leptospirosis | Listeriosis | Salmonellosis | Spotted fevers | Listeriosis |
| 13 | Listeriosis | Leptospirosis | Zoonotic tuberculosis | Leptospirosis | Tularemia | Tetanus | Leptospirosis |
| 14 | Leptospirosis | Listeriosis | Bovine cysticercosis | Zoonotic tuberculosis | Q-fever | Leishmaniasis | Zoonotic tuberculosis |
| 15 | Zoonotic tuberculosis | Tularemia | Hydatidosis | Bovine cysticercosis | Orf | MERS | Bovine cysticercosis |

^#^Final prioritized disease list for Uganda was selected by group discussion after overall results presented
